# Evolutionary history of relict *Congeria* (Bivalvia: Dreissenidae): unearthing the subterranean biodiversity of the Dinaric Karst

**DOI:** 10.1186/1742-9994-10-5

**Published:** 2013-02-06

**Authors:** Helena Bilandžija, Brian Morton, Martina Podnar, Helena Ćetković

**Affiliations:** 1 Division of Molecular Biology, Rudjer Boskovic Institute, Bijenička 54, 10 000 Zagreb, Croatia; 2 Croatian Biospeleological Society, Demetrova 1, 10 000 Zagreb, Croatia; 3 Department of Zoology, The Natural History Museum, Cromwell Road, London SW7 5BD, UK; 4 Croatian Natural History Museum, Demetrova 1, 10 000 Zagreb, Croatia

**Keywords:** Dinaric Karst, Subterranean habitats, Cave bivalve, *Congeria*, Dreissenidae, New species, Ecophenotypic plasticity

## Abstract

**Background:**

Patterns of biodiversity in the subterranean realm are typically different from those encountered on the Earth’s surface. The Dinaric karst of Croatia, Slovenia and Bosnia and Herzegovina is a global hotspot of subterranean biodiversity. How this was achieved and why this is so remain largely unresolved despite a long tradition of research. To obtain insights into the colonisation of the Dinaric Karst and the effects of the subterranean realm on its inhabitants, we studied the tertiary relict *Congeria*, a unique cave-dwelling bivalve (Dreissenidae), using a combination of biogeographical, molecular, morphological, and paleontological information.

**Results:**

Phylogenetic and molecular clock analyses using both nuclear and mitochondrial markers have shown that the surviving *Congeria* lineage has actually split into three distinct species, i.e., *C. kusceri, C. jalzici* sp. nov. and *C. mulaomerovici* sp. nov., by vicariant processes in the late Miocene and Pliocene. Despite millions of years of independent evolution, analyses have demonstrated a great deal of shell similarity between modern *Congeria* species, although slight differences in hinge plate structure have enabled the description of the two new species. Ancestral plesiomorphic shell forms seem to have been conserved during the processes of cave colonisation and subsequent lineage isolation. In contrast, shell morphology is divergent within one of the lineages, probably due to microhabitat differences.

**Conclusions:**

Following the turbulent evolution of the Dreissenidae during the Tertiary and major radiations in Lake Pannon, species of *Congeria* went extinct. One lineage survived, however, by adopting a unique life history strategy that suited it to the underground environment. In light of our new data, an alternative scenario for its colonisation of the karst is proposed. The extant *Congeria* comprises three sister species that, to date, have only been found to live in 15 caves in the Dinaric karst. Inter-specific morphological stasis and intra-specific ecophenotypic plasticity of the congerid shell demonstrate the contrasting ways in which evolution in the underground environments shapes its inhabitants.

## Background

Subterranean habitats are often colonised, either actively or passively, by unusual and highly distinctive animals, which in many cases are remnants of the surface fauna that once lived above them. These animals are often referred to as living fossils, or relict species. *Congeria kusceri* Bole, 1962, the only known troglobiotic bivalve [1], is a good example of this.

During the Tertiary, most of Europe was covered by a vast aquatic ecosystem of swamps and lakes spreading from the Swiss molasse Basin to Lake Aral in Central Asia. Within this system, known as the Paratethys, a spectacular radiation of many molluscs and other animal taxa occurred [2]. Here, the Dreissenidae Gray, 1840, a family of freshwater bivalves, flourished and diversified [3]. All of the five dreissenid genera [4] evolved in the Neogene lake systems of the Paratethys but only three have survived until the present day: *Mytilopsis* Conrad, 1857, *Dreissena* van Beneden, 1835 and *Congeria* Partsch, 1835. Many different *Congeria* species inhabited the Paratethys. Harzhauser & Mandic [3] identified 16 species and 11 subspecies, while Kochansky-Devide & Sliskovic [5] identified ~30 species from Miocene deposits in Croatia and Bosnia and Herzegovina alone. By the end of the Miocene, however, all but one had become extinct. *Congeria kusceri*, the only species known to have survived this dramatic period, is restricted today to but a few caves in the Dinaric Karst.

The Dinaric Karst extends for about 56,000 km^2^ along an 800 km arc from Trieste, Italy in the north, throughout most of Slovenia (SI), Croatia (HR) and Bosnia and Herzegovina (BA) to Albania in the south and is intersected by a network of caves, pits, underground lakes, rivers and streams containing one of the most complex and diverse subterranean faunas in the world [6,7]. It has been argued that the causes of such high subterranean biodiversity in the Dinarides lie in its complex geological history and intensive karstification that enabled multiple entries into the subterranean realm [8]. *Congeria* species, unlike other cave animals, have a rich fossil record, and can provide new insights into the timeframe, sources and causes leading to the biodiversity hotspot within the Dinaric Karst.

*Congeria kusceri* was first discovered in the 1930’s in deposits of empty shells, but a living population was not found until 20 years later in Žira cave in Popovo polje, southern Herzegovina, allowing J. Bole to describe the species in 1962. Later, additional living populations were found in distant areas of the Dinarides [9,10]. Recent extensive field researches have resulted in the discovery of a total of 15 known *Congeria* populations (Jalžić & Bilandžija, unpublished). Because of such a small number of sites, habitat destruction, and declines in population numbers, the species is listed as vulnerable (VU) in the Red List of European freshwater molluscs [11] and, in Croatia, *C. kusceri* is assessed as critically endangered (CR) [12].

It is currently assumed that there is only one species of stygobiotic bivalve - *Congeria kusceri -* that has a wide, holodinaric, distribution. Subterranean habitats are, however, subjected to fragmentation, leading potentially to lineage isolation and speciation. Conversely, the extreme character of the subterranean karst environment drives convergent adaptations in its inhabitants, resulting in cryptic morphologies and possibly masking real diversities [13,14]. Accordingly, widely-distributed cave animals have split into a number of lineages with small fragmented ranges [15], as demonstrated by molecular studies of several groundwater Dinaric taxa – the olm, *Proteus anguinus* Lorenti, 1768 [16], the cave shrimp, *Troglocaris*[17,18] and the water louse *Asellus aquaticus* (Linnaeus, 1758) [19].

In this study we have gathered biogeographical and paleontological data and used both molecular and morphological analyses to address several questions. We deal with contentious issues regarding the phylogenetic position and affinities of *Congeria* within the Dreissenidae. We have examined the question of *Congeria* lineage diversifications in separate parts of the Dinaric Karst, and explored the evolutionary history that ultimately caused a shift, uniquely amongst bivalves, towards a subterranean way of life. Finally, we have reported the effects of the underground environment on *Congeria* shell morphology. For the first time, therefore, this study combines several approaches to provide a new understanding of the evolutionary biology of *Congeria* and uncovers speciation events leading to the description of new *Congeria* species.

## Results

### Biogeography

**Figure 1:**
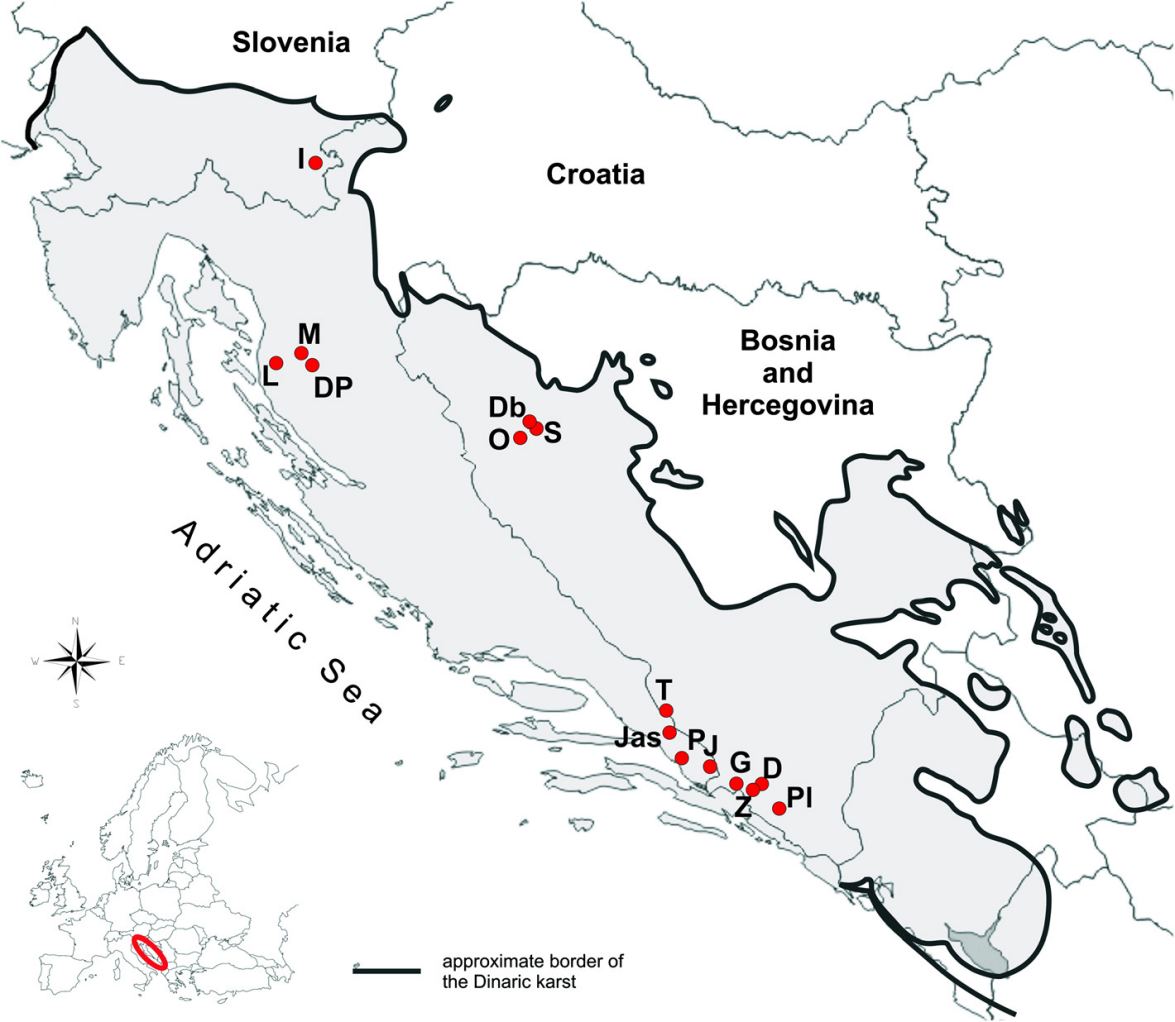
**A map of the Dinaric Karst showing all known localities where living populations of *Congeria *occur.** (**i**). Bela Krajina Region, (SI): I, Izvir Jamske Školjke. (**ii**). Lika Region, (HR): M, Markov Ponor; L, Lukina jama–Trojama Cave System; Dp, Dankov Ponor. (**iii**), north-western Bosnia, (BA): O, Oko; S, Suvaja; Db, Dabarska Pećina. (**iv**), Neretva Basin, southern Dalmatia (HR) and Herzegovina (BA): T, Tihaljina; Jas, Jasena Ponor; P, Pukotina u Tunelu Polje Jezero–Peračko Blato; J, Jama u Predolcu; G, Gradnica; Z, Žira; D, Doljašnica; Pl, Plitica.

*Congeria* is restricted to only 15 caves (Figure 1) which belong to four geographic regions, each hydrologically isolated from the others: one is in the Kupa River basin, Bela Krajina region (SI), three in the Lika River basin, Lika region (HR) and three in the Sana River basin in north-western Bosnia (BA). The remaining eight populations occur in the Neretva River basin in southern Dalmatia (HR) and Herzegovina (BA).

### Phylogenetic analyses

The final dataset consisting of all four concatenated gene markers contained 3847 nucleotides. Of these, 1033 sites were variable and 636 were parsimony informative. MP analysis resulted in 294 equally parsimonious trees (length 1598). Five runs of ML analysis computed in Garli resulted in the same topology, and the log-likelihood scores were similar in each run. Independent Bayesian runs converged to the stationary distribution. Inspection in Tracer showed acceptable ESS (effective sample size) values and good mixing of chains.

**Figure 2:**
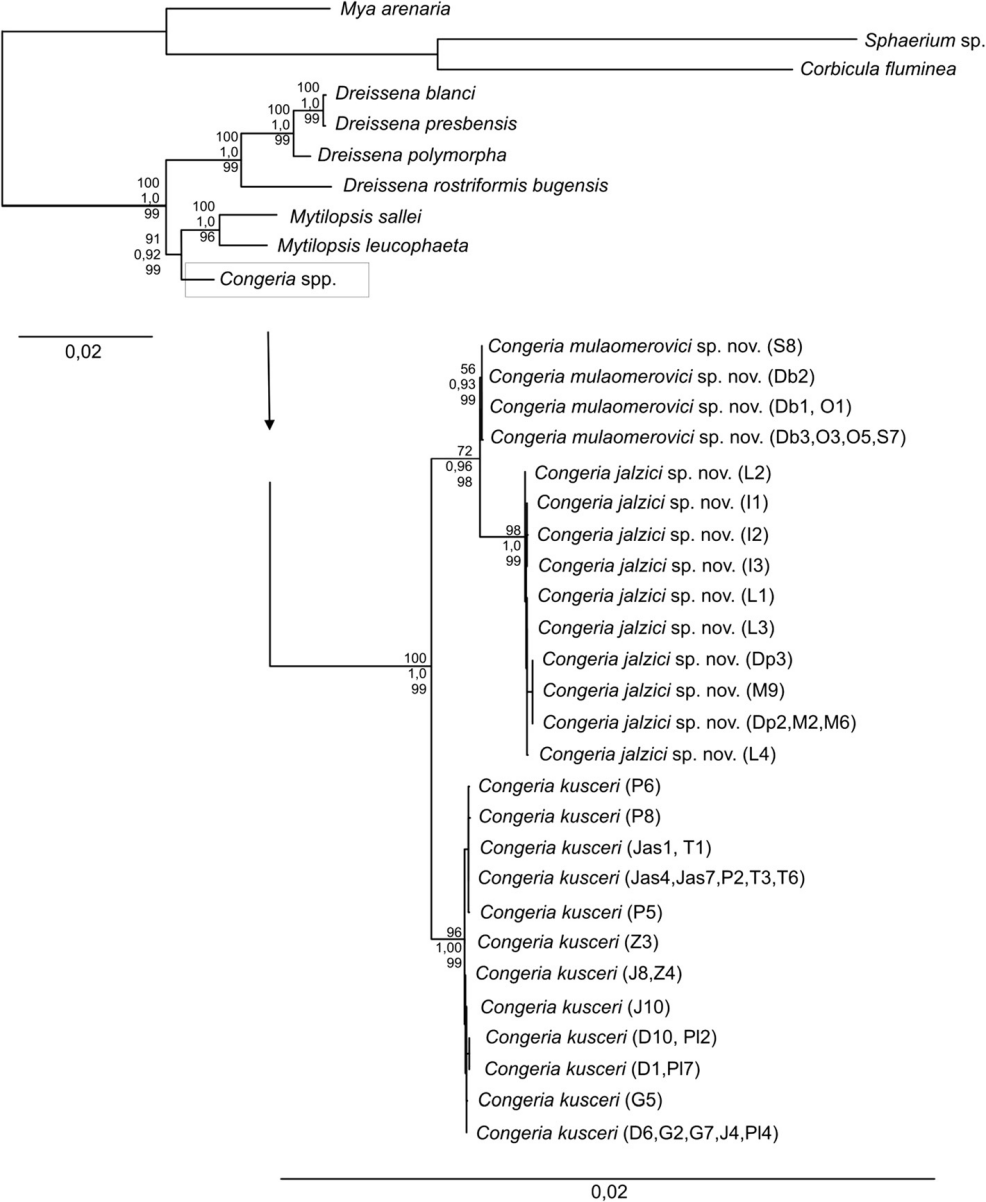
**Maximum likelihood phylogram based on combined nuclear (*18S *and *28S rRNA*) and mitochondrial (*COI *and *16S rRNA*) gene fragments.** Numbers on the nodes indicate ML bootstrap values (uppermost value), Bayesian posterior probabilities (in the middle) and MP bootstrap values (lowest value). Abbreviations next to *Congeria* branches stand for localities. See Figure 1 legend for locality abbreviations.

MP, ML and Bayesian concatenated trees were well resolved with most main branches showing high statistical support (>95% MP and ML bootstrap values and >98% BPP). Lower bootstrap and posterior probabilities were associated with the north-western Bosnian populations, probably due to lower resolution in the sequences at this shallow phylogenetic level (Figure 2).

All genetic markers and phylogenetic reconstruction methods employed supported the Dreissenidae as well as the three extant genera as monophyletic clades. Within the family, the first split isolated *Dreissena,* leaving *Mytilopsis* and *Congeria* as sister groups. The only exceptions to this overall topology were due to conflicting phylogenetic signals in the *16S* and *18S rRNA* trees, but without good statistical support.

*Congeria* was, thus, always monophyletic and divided into three subclades, although in some cases not with high support. Each of the subclades was restricted to a distinct geographic region: one subclade comprised populations from the Lika and Kupa River basins, another comprised individuals from the Sana River basin and the third comprised all southern populations from the Neretva River basin.

### Divergence dating

**Figure 3:**
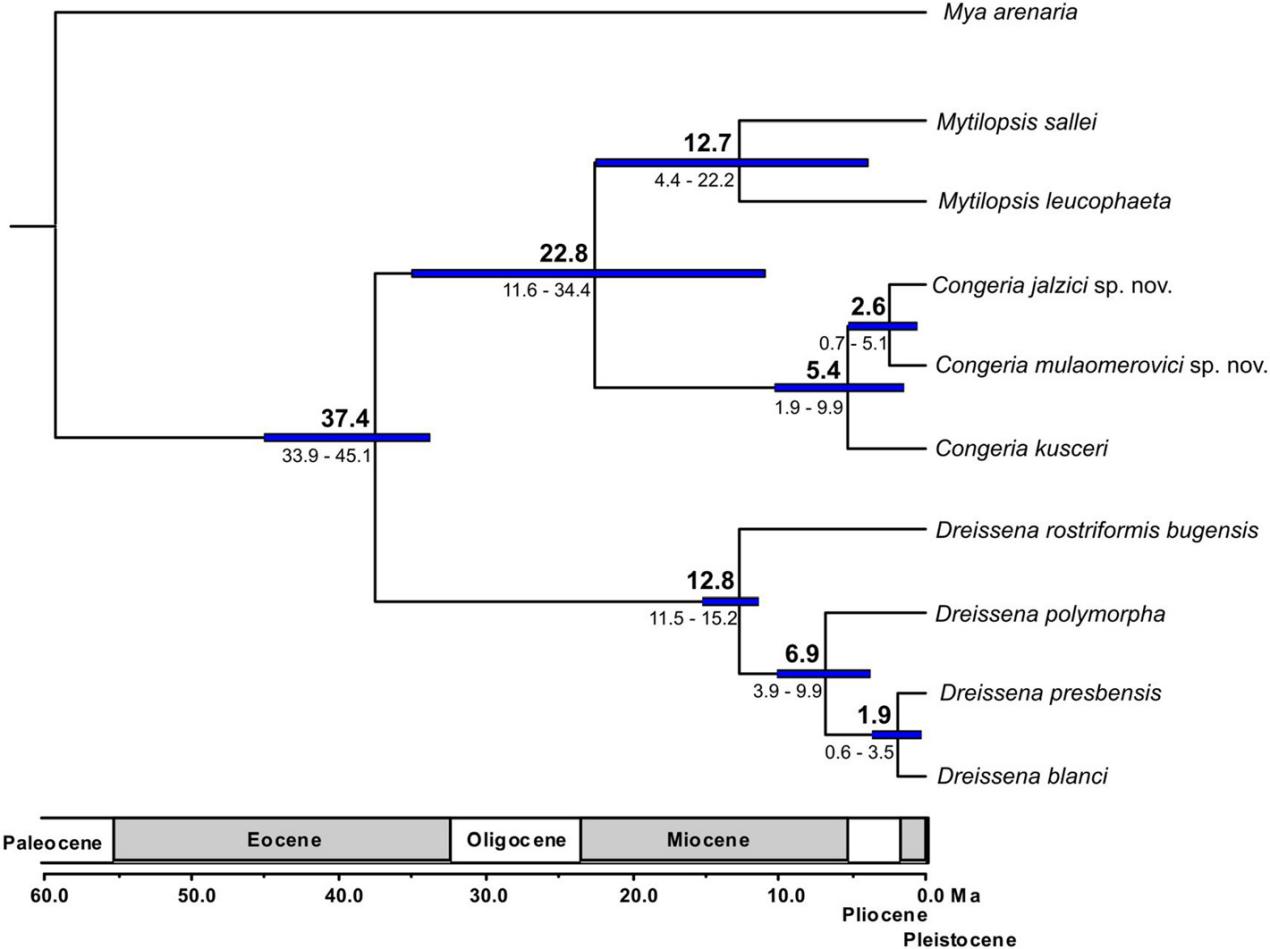
**Age estimates of evolutionary divergence events within Dreissenidae.** Maximum clade credibility chronogram based on BEAST analysis (lognormal clock model) of concatenated sequences of four genes (*18S*, *28S* and *16S rRNA* and *COI*). Mean divergence ages are shown above the nodes and 95% highest posterior density intervals (95% HPD ) are given in parentheses below nodes and denoted by blue horizontal bars. Major geological periods are indicated in million years on the time scale bellow the tree. Bayesian posterior probabilities were 1.0 for all nodes except for *Mytilopsis* + *Congeria* node (0.99) and *C. jalzici* + *C. mulaomerovici* node (0.99).

Separate molecular clock analyses using either the lognormal or exponential clock models, different prior distributions on the mean of the branch rates and on calibration nodes gave concordant divergence times in all but one instance (see below), demonstrating that the results are robust and not dominated by the choice of models and priors. The crown node of the family was estimated at 37.4 million years ago (MYA) (mean node age), which corresponds to the Priabonian Age and the occurrence of the first identifiable dreissenid fossils. The timing of the first split within *Dreissena* was set with lognormal prior placing a minimum hard bound at 11.6 MYA, when the genus first appeared in the fossil record. Accordingly, the first split, between *D. rostriformis bugensis* and the remaining three *Dreissena* spp., was estimated at 12.7 MYA. *Dreissena polymorpha* branched off next at 6.9 MYA. Finally, *D. blanci* and *D. presbensis* separated at 1.9 MYA (Figure 3).

The split between *Congeria* and *Mytilopsis* lineages was estimated to have occurred between 22.6 MYA, and the two *Mytilopsis* species split between 12.7 MYA. The estimates of the splits within *Congeria* differed according to the molecular clock model used. The exponential clock model placed these divergence events deeper in the past than the lognormal clock model did.

### Descriptions of the new species

DREISSENOIDEA Gray in Turton, 1840

Dreissenidae Gray in Turton, 1840

***Congeria*** Partsch, 1835

Type species, *Congeria subglobosa* Partsch, 1835, subsequent designation by Pilsbry, 1911. [=*Enocephalus* Münster, 1831 (*nomen nudum*)].

**Figure 4:**
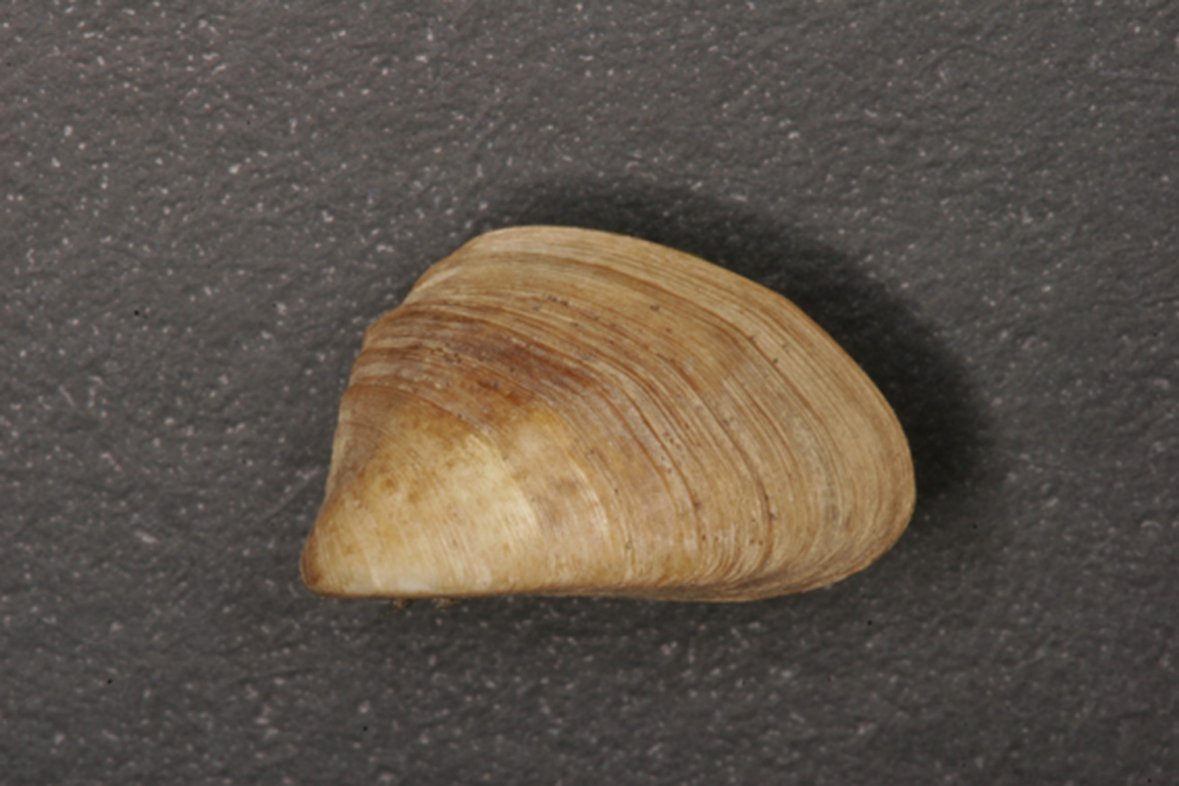
***Congeria jalzici *sp. nov.** The holotype from Markov Ponor, Lipovo polje, Lika, Croatia. Croatian Natural History Museum, Zagreb, Croatia (Reg. No.: 10346).

***Congeria jalzici *sp. nov. Morton & Bilandžija, 2013**. (Figures 4, 6A and 6B, 7A and D)

### Material examined

HOLOTYPE. General Collection of Recent Molluscs, Croatian Natural History Museum, Zagreb (CNHM, Reg. No.: 10346). Locality: Markov Ponor, Lipovo Polje, Lika, Croatia (Co-ordinates: WGS84 x = 44°45^′^57″: y = 15°10^′^53″). Leg: B. Jalžić and H. Bilandžija, 2008–2009. Shell length: 11.7 mm; height: 7.0 mm; width: 8.0 mm (Figure 4).

PARATYPES: Specimens 1–3, General Collection of Recent Molluscs, Croatian Natural History Museum, Zagreb (CNHM, Reg. No.: 10347); Specimens 4–6, The Natural History Museum, London (Reg. No’s.: NHMUK 20110180–20110182). Locality: Markov Ponor, Lipovo Polje, Lika, Croatia (Co-ordinates: WGS84 x = 44°45^′^57″: y = 15°10^′^53″). Leg: B. Jalžić and H. Bilandžija, 2008–2009 (Table 1).

**Table 1:** **Shell dimensions of *Congeria jalzici *sp. nov.**

**Shell length**	**Shell height**	**Shell width (mm)**
11.4	6.7	6.8
10.2	5.9	6.8
11.1	6.6	6.9
11.5	6.4	7.3
11.0	6.7	7.1
10.7	6.3	6.9

VOUCHER MATERIAL: Specimens 1 & 2, ecophenotypes of *Congeria jalzici* sp.nov.: General Collection of Recent Molluscs, Croatian Natural History Museum, Zagreb (CNHM, Reg. No.: 10348); Specimens 3 & 4, The Natural History Museum, London (Reg. No’s.: NHMUK 20110183 & 20110184). Locality: Lukina Jama – Trojama Cave System, Northern Velebit, Lika, Croatia. (Co-ordinates: WGS84 x = 44°46^′^04″: y = 15°01^′^52″). Leg: B. Jalžić, 2010 (Table 2).

**Table 2:** **Shell dimensions of *Congeria jalzici *sp. nov. ecophenotypes**

**Shell length**	**Shell height**	**Shell width (mm)**
11.6	7.6	6.0
12.4	7.4	6.7
11.5	7.3	6.2
10.9	6.7	6.4

### Description

Shell small, up to 13 mm in length, approximately equivalve, and distinctly inequilateral. Shell generally wider than it is tall, but often only slightly so. Periostracum brown. Distinctly heteromyarian with the swollen posterior face generally round; anterior narrowly rounded with the beaks pointed downwards. Postero- and antero-ventrally convex, although typically concave mid to antero-ventrally around a distinct byssal notch. Valve margins uniform, except ventrally around byssal notch where they are sinusoidal to varying degrees. An external, opisthodetic, ligament. Anterior adductor muscle scar situated on a small septum whose internal face is characteristically and smoothly rounded. Apophysis tiny, situated dorsal to the septum and located (partially hidden) under the resilifer and bears the tiny scar of the anterior byssal retractor muscle.

### Remarks

As with its sister species, *Congeria kusceri*, the shell of *Congeria jalzici* sp. nov. is variable in form, but the septum is small and distinctively concave. Hence, the anterior adductor muscle scar of the former is much larger and has a near straight internal margin aligned with the straight septum margin.

The ecophenotype of *Congeria jalzici* sp. nov. from the Lukina Jama – Trojama Cave System is different, in terms of shell form, from the specimens obtained from the type locality. It has a near transparent shell, with the periostracum only obvious as a yellow – light brown marginal fringe. Its internal shell septum is even smaller than that of conspecifics from Markov Ponor and the shell has a less triangular form in cross-section.

### Etymology

*Congeria jalzici* sp. nov. is named after Branko Jalžić, Croatian Natural History Museum, in honour of his achievements in the field of cave biology in the Dinarides and in appreciation of his invaluable help during this research.

**Figure 5:**
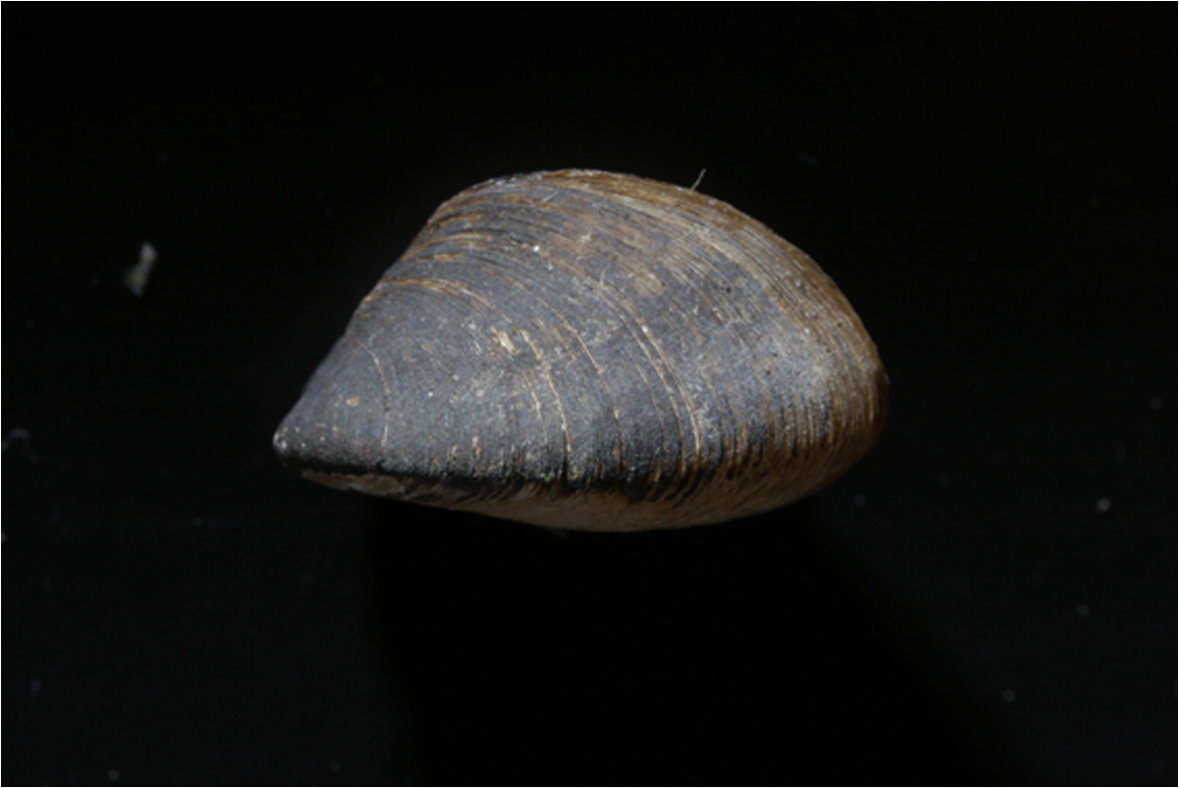
***Congeria mulaomerovici *sp. nov.** The holotype from Oko, Lušci Palanka, north-western Bosnia, Bosnia and Herzegovina. The National Museum of Bosnia and Herzegovina, Sarajevo, Bosnia and Herzegovina (Reg. No.: 470).

**Figure 6:**
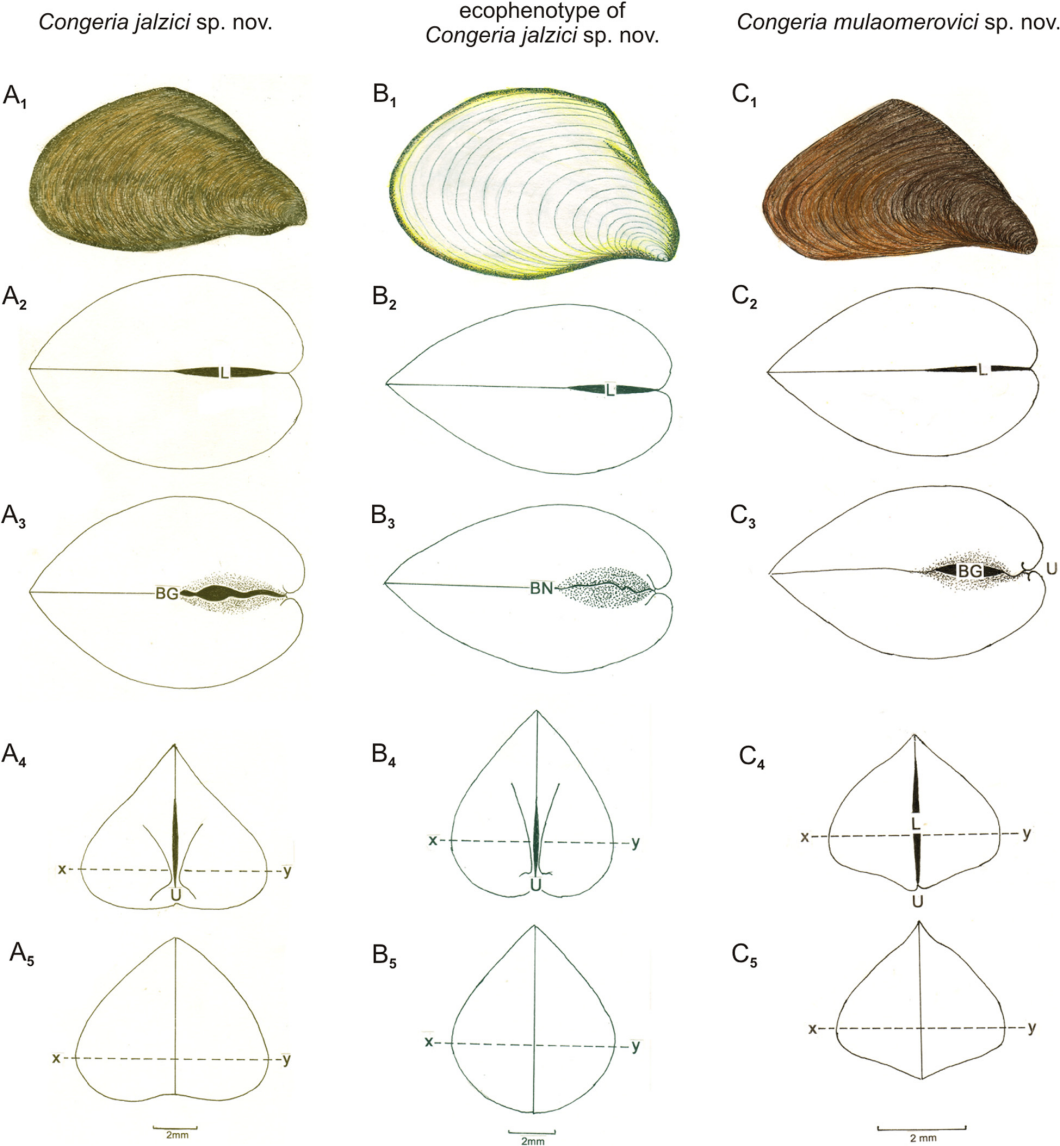
**Shells of *Congeria.*** A shell of (**A**) *Congeria jalzici* sp. nov. from Markov Ponor, (**B**) the ecophenotype of *Congeria jalzici* sp. nov. from the Lukina Jama-Trojama Cave System, and (**C**) *Congeria mulaomerovici* sp. nov. from Oko. 1, right lateral view; 2, dorsal view; 3, ventral view; 4, anterior view; 5, posterior view. (x---y indicates the greatest shell width). Abbreviations: BG - Byssal gape; BN - Byssal notch; L - Ligament; U - Umbo. x-----y equals the greatest width of the shell.

**Figure 7:**
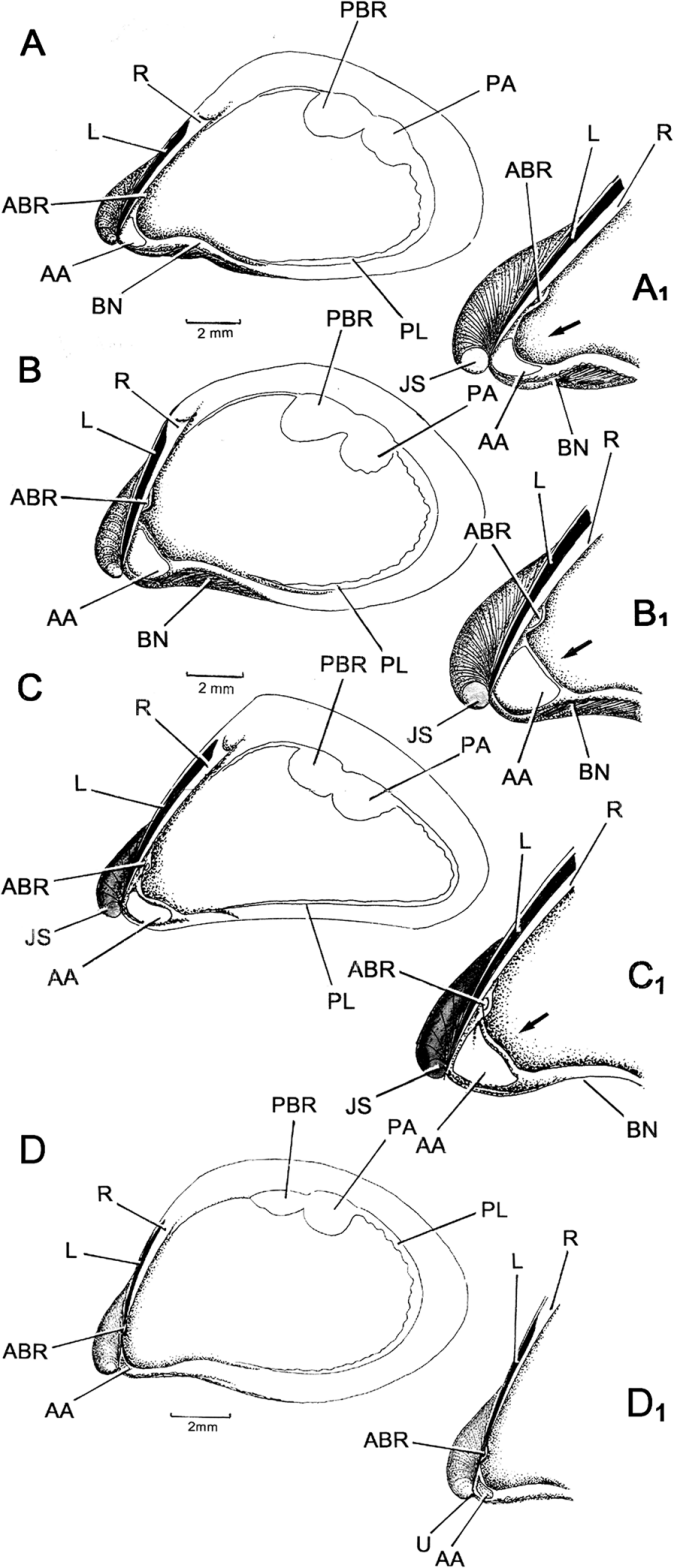
**Internal shell structures.** Internal views of the shells of **A**, *Congeria jalzici* sp. nov., **B**, *Congeria kusceri*, **C**, *Congeria mulaomerovici* sp. nov., and **D**, the ecophenotype of *Congeria jalzici* sp. nov. from the Lukina Jama-Trojama Cave System. **A**_ı_, **B**_ı_, **C**_ı_, and **D**_ı_ are details of the hinge plates of the four specimens with the arrows pointing to the septa on which is inserted the anterior adductor muscles. Abbreviations: AA - Anterior adductor muscle scar; ABR - Anterior retractor muscle scar; BN - Byssal notch; JS - Juvenile shell; L - Ligament; PA - Posterior adductor muscle scar; PBR - Posterior byssal retractor muscle scar; PL - Pallial line; R - Resilifer; U - Umbo.

***Congeria mulaomerovici *sp. nov. Morton & Bilandžija, 2013**. (Figures 5, 6C, 7C)

### Material examined

HOLOTYPE. Collection of Molluscs, The National Museum of Bosnia and Herzegovina, Sarajevo (Reg. No.: 470). Locality: Oko, Lušci Palanka, north-western Bosnia, Bosnia and Herzegovina (Co-ordinates: WGS84 x = 44°42^′^08″: y = 16°28^′^04″). Leg: B. Jalžić, 2011. Shell length: 11.8 mm; height: 6.8 mm; width: 8.0 mm (Figure 5).

PARATYPES: Specimens 1–3, The National Museum of Bosnia and Herzegovina, Sarajevo (Reg. No.: 471); Specimens 4–6, The Natural History Museum, London (Reg. No’s.: NHMUK: 20110469/1,2,3); Specimens 7–9, General Collection of Recent Molluscs, Zoology Department, Croatian Natural History Museum, Zagreb (CNHM, Reg. No.: 10348). Locality: Oko, Lušci Palanka, Bosnia and Herzegovina. Leg: B. Jalžić, 2011 (Table 3).

**Table 3:** **Shell dimensions of *Congeria mulaomerovici *sp. nov.**

**Shell length**	**Shell height**	**Shell width (mm)**
12.0	7.3	7.6
10.9	7.2	6.6
10.8	6.7	6.3
10.6	6.7	6.4
11.2	7.1	7.1
10.4	6.1	6.7
10.2	6.2	6.1
10.4	6.4	6.5
10.0	6.2	6.0

### Description

Shell small, up to 12 mm in length, approximately equivalve but distinctly and acutely inequilateral. Shell usually wider than it is tall, but often only slightly so. Periostracum uniformly brown. Distinctly heteromyarian with the postero-dorsal slope straight and, hence, sharply pointed; anteriorly also pointedly rounded. Ventrally flattened, although somewhat concave antero-ventrally around a slight byssal notch. Valve margins uniform, except ventrally around the byssal notch where they are slightly sinusoidal to varying degrees. The beaks point downwards. An external, opisthodetic, ligament. Anterior adductor muscle scar situated on a small septum whose internal face is smoothly sinusoidal. Apophysis small, situated dorsal to the septum and located (partially hidden) under the resilifer and bears the tiny scar of the anterior byssal retractor muscle.

### Remarks

As with its sister species, *Congeria kusceri* and *Congeria jalzici* sp. nov., the shell of *Congeria mulaomerovici* sp. nov. is variable in overall form but is more distinctively pyramidal dorsally. Further, the septum is sinusoidal, such that the anterior adductor muscle scar is bean-shaped.

### Etymology

*Congeria mulaomerovici* sp. nov. is named after Dr. Jasminko Mulaomerović, Centre for Karst and Speleology, Sarajevo, an eminent researcher of the karst in Bosnia and Herzegovina, and in appreciation of his support during our research.

### A comparison of shell form

*Congeria jalzici* sp. nov. Seen from the right (Figure 6A_1_), the shell is antero-dorsally keeled and deeply convex at the midpoint around the keel. Seen from the dorsal aspect (Figure 6A_2_), the shell is posteriorly pointed and laterally inflated. The ventral valve margins (Figure 6A_3_) are straight posteriorly, anteriorly they are sinusoidal around a large byssal gape (BG). The separated umbones (U) are clearly obvious when seen from the anterior aspect (Figure 6A_4_). The shell is flattened ventrally and the greatest shell width (x---y) is situated close to the ventral side making the shell of *C. jalzici* sp. nov. distinctly mytiliform. From the posterior aspect (Figure 6A_5_), the shell is more rounded laterally and concave centrally.

Ecophenotype of *Congeria jalzici* sp. nov.. The shell of *C. jalzici* sp. nov. from the Lukina Jama – Trojama Cave System is distinctly less antero-dorsally keeled than conspecifics from the type locality (Figure 6B_1_) and therefore less concave at the mid antero-dorsal point. It is also posteriorly more rounded, less anteriorly convex and concave postero-ventrally around the byssal notch. Seen from the dorsal and ventral aspect (Figure 6B_2_, 6B_3_), the shell is like its type locality conspecifics except there is not a byssal gape although there is a shallow byssal notch (BN). In cross-section (Figure 6B_4_), the left and right valves are not indented as in type conspecifics but are more smoothly rounded to create a more drop-shaped form. The shell is less flattened ventrally, except at the valve margins, which are concave anteriorly. The greatest shell width (x---y) is situated at a point more dorsally than in type locality conspecifics and thus is not mytiliform. From the posterior aspect (Figure 6B_5_), the shell is distinctly rounded laterally and is not flattened ventrally as in type locality conspecifics. The shell of this population of *C. jalzici* sp. nov. is clearly not adapted to flowing waters as are conspecifics from the type locality.

*Congeria kusceri*. The shell of *Congeria kusceri* has been described by Morton *et al.* (1998, Figures seven-sixteen).

*Congeria mulaomerovici* sp. nov.. The shell of *C. mulaomerovici* sp. nov. is somewhat antero-dorsally keeled but at a point more anteriorly than in *C. jalzici* sp. nov. (Figure 6C_1_). It is dorsally peaked, almost pyramidal. Seen from the dorsal aspect (Figure 6C_2_), the shell is posteriorly pointed and laterally inflated. The ventral valve margins (Figure 6C_3_) are slightly curved posteriorly, anteriorly they are somewhat sinusoidal around a large byssal gape (BG). The right valve overlaps the left somewhat posterior and, especially, anteriorly such that the umbones (U) are distinctly unequally situated, the left more anterior than the right. The umbones are also less separated than in *C. jalzici* sp. nov.. The valves are slightly laterally indented in cross-section (Figure 6C_4_, 6C_5_) and the shell is ventrally keeled. As a consequence, the greatest shell width (x---y) is situated more dorsally than in *C. jalzici* sp. nov. so that the whole form of the shell is less mytiliform.

### A comparison of hinge plates

*Congeria jalzici* sp. nov. (Figure 7A). Internally, the shell possesses a large posterior adductor muscle scar (PA), internal to which is the scar of the posterior byssal retractor muscle (PBR). There is a thick pallial line (PL), especially posteriorly and a small, bean-shaped anterior adductor muscle scar (AA) located on a shell shelf or septum, internal to the downwardly directed umbones. The long thin ligament is situated on a resilifer and extends approximately half way up the anterior slope of the shell. Underneath the resilifer, just above the shell shelf is a tiny apophysis on which is located the scar of the anterior byssal retractor muscle (ABR). There is a deep byssal notch (BN). The shell shelf (Figure 7A_1_) has a distinctively curved inner margin (arrowed).

The ecophenotype of *Congeria jalzici* sp. nov. (Figure 7D). The shell is altogether more delicate than in *C. jalzici* sp. nov. from its type locality. Similarly, the internal muscle scars are smaller and more delicate – indeed they are difficult to discern in such a thin, near-translucent, shell but their arrangement is approximately the same. In the ecophenotype of *C. jalzici* sp. nov., the shell septum is extremely delicate, but has the same form, a distinctively curved inner margin (Figure 7D_1_). The scar of the tiny anterior adductor muscle (AA) is located just internal to the umbo (U). The apophysis with its scar of the anterior byssal retractor muscle (ABR) is similarly proportionally smaller than in *C. jalzici* sp. nov. from its type locality.

*Congeria kusceri* (Figure 7B). The arrangement of the internal muscle scars are approximately the same as in *C. jalzici* sp. nov.. In *C. kusceri*, however, the shell septum is proportionally larger than in *C. jalzici* sp. nov., as are the scars of the anterior adductor muscle (AA) and the anterior byssal retractor muscle (ABR) situated on its also proportionally larger apophysis. The shell septum of *C. kusceri* (Figure 7B_1_) has a straight inner margin (arrowed) and the apophysis is located much closer to the shell septum.

*Congeria mulaomerovici* sp. nov. (Figure 7C). The shell is more steeply pointed dorsally and is distinctively more pointed posteriorly than in both *C. kusceri* and *C. jalzici* sp. nov., but the byssal notch is small. The arrangement of the internal muscle scars is approximately the same as in the previous two species. In *C. mulaomerovici* sp. nov., however, the shell septum is approximately mid way in size between the other two *Congeria* species as is the scar of the anterior adductor muscle (AA). The apophysis with its scar of the anterior byssal retractor muscle (ABR) is approximately the same size as in *C. kusceri* but, of all the three species it is located the closest to the shell septum and, in fact, partially beneath it (Figure 7C_1_). The shell shelf has a sinusoidal inner margin (arrowed).

## Discussion

The Dreissenidae is an excellent candidate group to study evolutionary processes that shape close relatives into biologically and ecologically diverse sets of species. Here we have focused on the most rare and exceptional taxon in the family - *Congeria* - a Tertiary relict that underwent significant changes in morphology, biology and ecology to be the only survivor of a once widespread and diverse genus.

### Phylogenetic relationships

Our results have shown that each of the three extant dreissenid genera form a monophyletic group. Although our *Mytilopsis* species representation is far from exhaustive, this confirms previous taxonomic understandings [20]. The sister relationship of *Congeria* and *Mytilopsis* is evident from both molecular and morphological characters. The presence of an apophysis is a common feature that separates them both from *Dreissena* but it has also been used as an argument to merge these two genera into one, that is, *Congeria*[21]. An apophysis is, however, the ancestral feature of the Dreissenidae and its sole use to infer dreissenid relationships has led to conclusions such as the polyphyletic origin of *Dreissena*. This view was most recently supported by Sket [22], who formally proposed the placement of *Congeria kusceri* into *Mytilopsis*. In a detailed study of the morphology of *C. kusceri*, however, Morton et al. [1] provided additional evidence to distinguish the species from others comprising *Mytilopsis*. Further, many aspects of the biology and ecology of *C. kusceri* are unique. In addition to its distinctive reproductive strategy, *C. kusceri* exhibits a wholly characteristic life history that involves extreme longevity (decades) [1] unlike the short lived (2–3 years), opportunistic, non-brooding, representatives of *Dreissena* and *Mytilopsis*[23,24]. Furthermore, our molecular clock analysis placed the timing of divergence between these two extant lineages at 22.6 MYA, arguing in favour of a special and distinctive placement for *Congeria.*

The results have demonstrated that instead of only one holodinaric species, as was previously thought, *Congeria* comprises at least three distinct species: *C. kusceri*, *C. jalzici* sp. nov. and *C. mulaomerovici* sp. nov.. Separate lineages have formed in the geographically and hydrologically isolated regions of the Dinaric Karst. Along with fragmentation of karstic underground habitats, both the sessile lifestyle and the reproductive strategy of *C. kusceri*[25] would not facilitate dispersal, so it is argued that speciation occurred after vicariant isolation of lineages in separate hydrological basins. Within the *C. jalzici* sp. nov. lineage, however, the isolated Slovenian population lacks any obvious genetic distinction, but shows slight and consistent differences in shell. Even if undiscovered populations exist between the Bela Krajina and Lika regions, it is unlikely that there is any gene flow present, because several hydrological basins and the divide between the Black Sea and the Adriatic Sea catchments separate these two populations. Without the possibility of communication, the most likely explanation for such a common genetic similarity would be a relatively recent split.

### Effects on morphology

**Figure 8:**
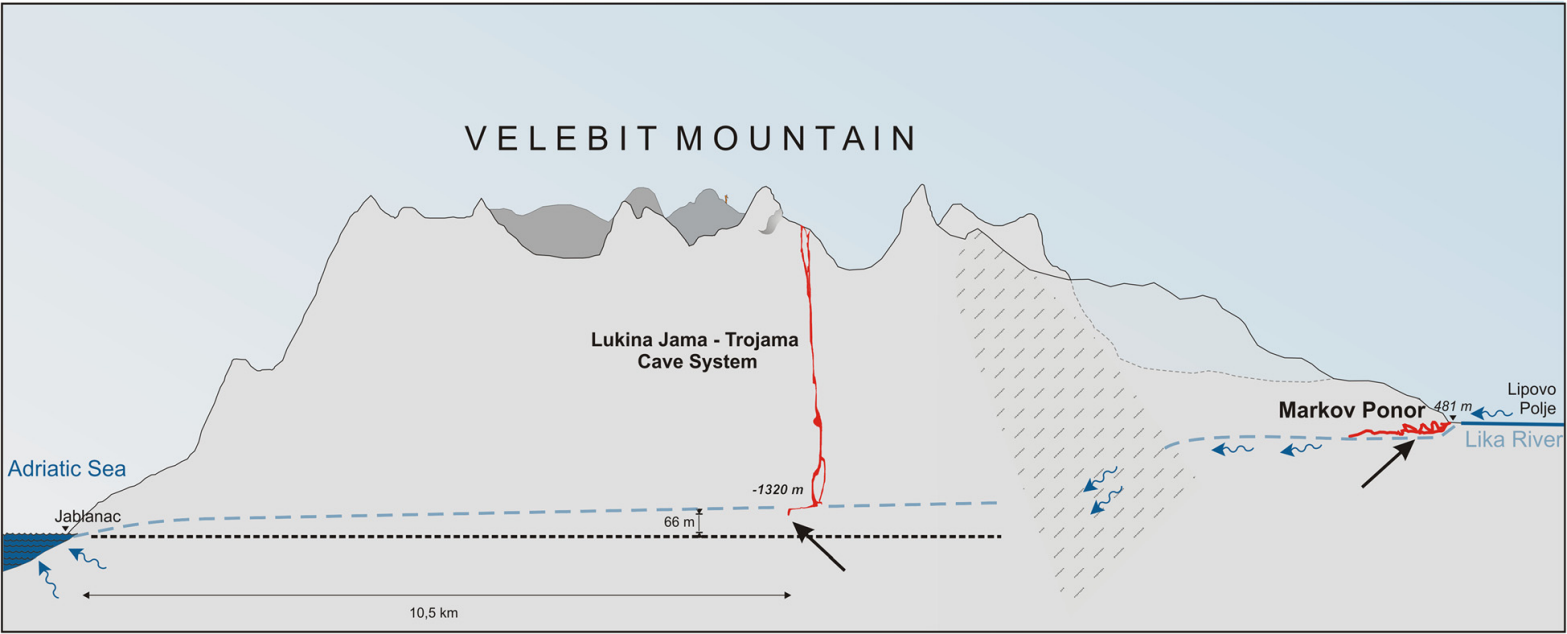
**A cross section through Velebit Mountain, Lika, Croatia.** The Markov Ponor and Lukina Jama – Trojama Cave System are hydrologicaly connected via underground conduits.

Over half of the known *Congeria* sites contain only empty shell deposits that were flushed to the surface by underground water currents. In order to be able to assign this material to any of the three *Congeria* species, we had to focus on finding distinctive shell characters. Shell morphometric measurements (Additional file 1) have, however, demonstrated that no single dimension differed significantly between all three species and the two species that are genetically and geographically most distant are most similar morphometrically. Shell morphology is, moreover, intra-specifically variable in all three species of *Congeria*, and *C. jalzici* sp. nov. is particularly remarkable in terms of a demonstrable shell plasticity. That is, two populations of this species, living in the same hydrological system (Figure 8), have significantly different shell morphologies. In the flowing waters of Markov Ponor, *C. jalzici* sp. nov. is characterised by a heteromyarian, ventrally flattened, shell whereas conspecifics from the Lukina Jama–Trojama Cave System at −1421 m below ground, are extremely delicate with ventrally concave shells and a reduced apophysis. In contrast to all other known *Congeria* localities where strong currents form during high water levels, the waters of the deep karst aquifer in the Lukina Jama–Trojama Cave System seem to be static and to rise and fall only slowly. The shape of *Congeria* shell and also the fact that tubes of *Marifugia cavatica* Absolon & Hrabe, 1930, grow perpendicular to the walls of the cave (B. Jalžić, personal information) point to this conclusion. The example of *C. jalzici* sp. nov. shows how the dreissenid shell is, to a great extent, shaped by environmental conditions and can be misleading, when viewed alone, in interpreting phylogenetic relationships within the family.

Finally, subtle differences between the three *Congeria* species have been identified in terms of hinge plate morphology. These include the sizes of shell septum and the anterior adductor muscle scar, the form of the inner margin and the position of the apophysis. These characters, although also showing variability, were consistent in all cave populations comprising one phylogenetic lineage (including the ecophenotype of *C. jalzici* sp. nov.). Interestingly, Schütt [21] distinguished a number of dreissenid species also on the detailed structure of the hinge plate/resilifer/apophysis. Despite the difference in opinion with regard to the generic placement of these taxa, it is evident from both Schütt’s and our studies that the only useful shell characters for distinguishing between these species of Dreissenidae relate to the hinge plate.

### Divergence dating and evolutionary history

Our molecular clock estimates of divergence events within the Dreissenidae differ from those published previously [20,26,27]. All previous studies have utilised a strict clock for the timing of speciation events. In recent years, however, new methods have been developed that account for rate variation and assume uncorrelated rates of evolution [28,29], and these have been applied in the present study.

Although a wide uniform prior of 33.9-55.8 MYA was used to calibrate the origin of the Dreissenidae, the posterior estimates narrowed the divergence time to 37.4 MYA. This age estimate corresponds to the time frame of the formation of the Paratethys Sea [30] as well as the occurrence of the first certain dreissenid fossils [31]. Our study was not designed for divergence dating within *Dreissena* and the species coverage of this genus is incomplete in our dataset (for associated problems see Wilke [32]), but there is some correlation between our *Dreissena* divergence estimates and those reported in studies of Stepien et al. [20,26,27]. Their divergence ranges vary slightly from study to study, but they collectively place the split between *D. rostriformis bugensis* and *D. polymorpha* at around 10–15 MYA, which is consistent with the fossil record and supports our calibration choices. Our estimates of the divergence of *D. polymorpha* and the Balkan *D. blanci/D. presbensis* clade at 6.9 MYA is more ancient then reported by Stepien et al. [27]. The occurrence of *D. polymorpha* in lower Pliocene deposits from the southernmost remnants of Lake Pannon [3]*,* suggest that this species followed the immigration route into the eastern Paratethys, presumably through the Dacian Basin [2], rather later. This implies that the common ancestor of the Balkan clade started migration towards the central Balkans during the late Miocene. Sometime during the Pliocene it settled in Lake Ohrid, which became the source of today’s *Dreissena* biodiversity in the region [33]. This is in accordance with the late Miocene/early Pliocene timeframe of changes in distributions of *Dreissena* lineages [27]. The divergence between the two Balkan clades of *Dreissena* happened after ancestors of *D. blanci* invaded the southern Ionian region from Lake Ohrid [33] around 1.9 MYA, according to our estimates.

Our results show a divergence time of 22.6 MYA for the split between the ancestors of the extant *Mytilopsis* species from the Americas and the ancestors of stygobiotic *Congeria* spp. This estimate roughly corresponds to the Oligocene/Miocene boundary and follows after the isolation and the establishment of the Paratethys as an independent biogeographic unit in Late Oligocene [2]. This is in accordance with Nutall [4], who proposed that founder populations of extant *Mytilopsis* lineages invaded the New World in the Late Oligocene. Another possible scenario is a gene flow blockade between *Mytilopsis* populations from both sides of the Atlantic as a result of the isolation of the Paratethys. Following this event, the New World *Mytilopsis* evolved independently of their relatives that remained in Europe. After the split with *Congeria,* that occurred in the long-lived Lake Pannon about 11.6 MYA [3], the European lineages of *Mytilopsis* became extinct.

According to the exponential clock model, the first split within the extant lineages of *Congeria* happened around 8 MYA, what approximately corresponds to the ages of the holodinaric groups of *Troglocaris* and *Proteus*, although 7.5-8.5 MYA was the lowest estimate of the inferred ranges for these taxa [34]. The lognormal clock gave different estimates and the discrepancy is probably due to the fact that the exponential model assumes most branch rates are small. Due to their generation times, reproductive strategies and pronounced adaptability that enabled high invasiveness, most dreissenids would not be expected to have slow mutation rates. The lognormal clock therefore probably gives better estimates. The lognormal clock estimate of 5.4 MYA corresponds approximately with the disappearance of *Congeria* from the fossil record, which coincided with large paleogeographical changes in the region involving the final disappearance of both Lake Pannon and the bordering Dinaride Lake System [3]. Although the Dinaride Lake System is more frequently suggested as the source of stygobiotic *Congeria* ancestors [1,22] the adjacent Lake Pannon offers another possible alternative.

Lake Pannon was a long-lasting lake where *Congeria* originated and went through an exceptional radiation [2,3]. Its disappearance gradually progressed from north to south, and by the late Miocene/early Pliocene it occupied only the extreme south of its previous range, an area bordering the Dinaric Karst. Part of the Lake Pannon fauna has survived to the present day by immigration into other regions before the lake finally vanished. For example, Lake Pannon is considered to be the source of the Ponto-Caspian fauna and it also had an impact on the fauna of Balkan lakes such as Lake Ohrid [2]. According to one of the biogeographical scenarios presented by Albrecht et al. [35], the ancestor of endemic Balkan *Dreissena* migrated from the Pannon basin into central Balkan lakes that are situated even further from Lake Pannon than the neighbouring Dinarides. The origin of *Asselus aquaticus* (Linnaeus, 1758) lineages is also considered to be the western Pannonian region, from where it colonised the rest of Europe including the Dinaric Karst around 4–5 MYA [36]. It is, therefore, possible that the ancestral stock of the subterranean *Congeria* lineages invaded the Dinaric Karst from Lake Pannon. If so, this must have happened prior to the first divergence event that separated today’s north-western and south-eastern lineages. Perhaps it coincided with the split of the “Dinaro-Caucasian” lineage of *Troglocaris* into Dinaric and Caucasian clades, which was estimated at 6–11 MYA [17], implying that there must have been a faunal interchange between the Dinaric water systems and the Paratethyan basins of Central Europe up to that time period.

The second divergence event occurred in the northern portion of the present distribution and separated the northern Bosnian populations of *Congeria mulaomerovici* sp. nov. from *Congeria jalzici* sp. nov. at about 2.5 MYA according to the lognormal clock. This occurred much later than the isolation of the northern Bosnian populations from the remaining *Proteus* (4.4-5.4 MYA) and *Troglocaris anophthalmus* (Kollar, 1848) lineages (3.7-5.3 MYA) [17,34]. Along with divergence time estimates of various Dinaric groundwater taxa, the phylogeographical patterns are incongruent as well [15,16,18,34]. The disparities may be the result of dissimilarities in biology of these animals and/or a complex geological history of the Dinaric Karst. Progressive karstification alters hydrological relationships over time and consequently the distributions of phylogenetic lineages are often not concordant with present day hydrological regimes, for example in *Asellus aquaticus*[19], *Troglocaris anophthalmus*[18] and *Congeria jalzici* sp. nov..

On the other hand, the disparities in the divergence estimates may be a result of availability and employment of different molecular clock methodologies. In comparison to the other groundwater Dinaric taxa, hard-shelled dreissenids have a rich fossil record that can possibly enable more reliable time divergence dating. Unfortunately, plasticity of the dreissenid shell makes linking of cave *Congeria* lineages with any of the fossil species highly speculative so the questions regarding the last surface ancestor and its colonisation of the underground remain to be answered. Further studies are needed to resolve these issues and to create an integrated picture of the processes that shaped the subterranean biodiversity of the Dinaric Karst.

## Conclusions

In conclusion, *Congeria* is a distinct member of the Dreissenidae that is separated from its closest extant relatives - *Mytilopsis* - by ~22-23 million years of independent evolution. The exact origin of the subterranean *Congeria* lineage is problematic because the innate plasticity of the dreissenid shell, as demonstrated in this study, does not allow the cave *Congeria* lineage to be related to the fossil dressenids of either the Lake Pannon or the Dinaride Lake Systems. Isolation in the Dinaric Karst underground has driven the speciation of the three allopatric lineages of *Congeria*: *C. kusceri*, *C. jalzici* sp. nov., and *C. mulaomerovici* sp. nov., herein identified morphologically and genetically. Inter-specific morphological stasis of shell forms has not been interrupted during the colonisation of caves or subsequent speciations. Instead, a plesiomorphic shell form has been retained and remained relatively unaffected by the millions of years that the tree species have spent in isolation from each other. The divergent shell form within a single lineage illustrated by the ecophenotype of *C. jalzici* sp. nov. has possibly arisen as an adaptation to a specific subterranean microhabitat.

The tasks of understanding the evolutionary history of *Congeria* spp., especially their origin and colonisation of the subterranean habitats as well as how genotypic and ecophenotypic components interact, provide new interdisciplinary challenges.

## Methods

### Taxon sampling and identification

Samples of *Congeria* from all 15 caves (Figure 1) known to harbour living populations have been examined. Individuals were partly collected between 2008 and 2011. Others were obtained from the Croatian Natural History Museum, and the University of Ljubljana. *Dreissena polymorpha* Pallas, 1771 was collected from Lake Jarun, Zagreb, Croatia*. Dreissena rostriformis bugensis* (Andrusov, 1897) was obtained from Ijsselmeer, Lelystadt, The Netherlands, and *Mytilopsis sallei* (Récluz, 1849) was obtained from Hong Kong, China.

### DNA extraction, amplification cloning and sequencing

The fragments of two mitochondrial (*COI* and *16S rRNA*) and two nuclear (*28S* and *18S rRNA*) genes were sequenced from one individual of *Dreissena polymorpha*, *Dreissena rostriformis bugensis, Mytilopsis sallei* and 44 different *Congeria* specimens from 15 locations including, for the first time, the type locality of *C. kusceri*, Žira ponor. DNA was extracted using DNeasy Blood & Tissue kit (Qiagen) or i-genomic DNA extraction kit (Intron). The primers, PCR reaction components and cycling conditions are indicated in Additional file 2. PCR products were separated by electrophoresis in 0.5 to 1.5% agarose gels, excised from the gel and purified using QIAquick Gel Extraction Kit (Qiagen) or MEGAquick-spin PCR & Agarose Gel DNA Extraction System (Intron). DNA fragments were cloned into either pGEM-T or pGEM-T Easy Vector Systems (Promega) or were sequenced directly using an ABI PRISM 3100 automatic sequencer (Applied Biosystem).

PCR products were sequenced on both strands and inspected manually for ambiguities. The resulting sequences have been deposited in GenBank. Sequences from *Dreissena blanci* (Westerlund, 1890), *Dreissena presbensis* Kobelt, 1915, *Mytilopsis leucophaeta* (Conrad, 1831) and three selected outgroups were retrieved from GenBank. Accession numbers are listed in Table 4. Outgroups were chosen according to Park & O’Foighil [37] and Taylor et al. [38]. Since there is no consensus regarding the group most closely related to the Dreissenidae, *Mya arenaria* Linnaeus, 1758, *Corbicula fluminea* (O.F. Müller, 1774) and *Sphaerium striatinum* (Lamarck, 1818) were selected as outgroups.

**Table 4:** List of species and GenBank Accession numbers of sequences used in this study

**Species**	**Source**	**Specimen**	***18S rRNA***	***28S rRNA***	***16S rRNA***	***COI***
*Congeria kusceri*	Plitica, Popovo polje, BA	Pl2	JX099472	JX099493	JX099451	JX099431
		Pl4		JX524681	JX524654	JX524708
		Pl7		JX524682	JX524655	JX524709
*Congeria kusceri*	Žira, Popovo polje, BA	Z3	JX099475	JX099496	JX099454	JX099434
		Z4		JX524686	JX524659	JX524713
*Congeria kusceri*	Doljašnica, Popovo polje, BA	D1		JX524664	JX524637	JX524692
		D6		JX524665	JX524638	JX524691
		D10	JX099460	JX099481	JX099439	JX099419
*Congeria kusceri*	Gradnica, Neum, BA	G2		JX524666	JX524639	JX524693
		G5	JX099461	JX099482	JX099440	JX099420
		G7		JX524667	JX524640	JX524694
*Congeria kusceri*	Jama u Predolcu, Metković, HR	J4		JX524669	JX524642	JX524696
		J8		JX524670	JX524643	JX524697
		J10	JX099464	JX099485	JX099443	JX099423
*Congeria kusceri*	Pukotina u Tunelu Polje Jezero-Peračko Blato, Ploče, HR	P2		JX524679	JX524652	JX524706
		P5		JX524680	JX524653	JX524707
		P6	JX099470	JX099491	JX099449	JX099429
		P8	JX099471	JX099493	JX099450	JX099430
*Congeria kusceri*	Jasena, Vrgorac, HR	Jas1	JX099465	JX099486	JX099444	JX099424
		Jas4		JX524671	JX524644	JX524698
		Jas7		JX524672	JX524645	JX524699
*Congeria kusceri*	Tihaljina, Ljubuški, BA	T1	JX099474	JX099495	JX099453	JX099433
		T3		JX524684	JX524657	JX524711
		T6		JX524685	JX524658	JX524712
*Congeria mulaomerovici* sp.nov.	Oko, Lušci Palanka, Northern Bosnia, BA	O1	JX099469	JX099490	JX099448	JX099428
		O3		JX524677	JX524650	JX524704
		O5		JX524678	JX524651	JX524705
*Congeria mulaomerovici* sp.nov.	Suvaja, Lušci Palanka, Northern Bosnia, BA	S7		JX524683	JX524656	JX524710
		S8	JX099473	JX099494	JX099452	JX099432
*Congeria mulaomerovici* sp.nov.	Dabarska Pećina, Sanski Most, Northern Bosnia, BA	DB1	JX099459	JX099480	JX099438	JX099418
		DB2		JX524660	JX524633	JX524687
		DB3		JX524661	JX524634	JX524688
*Congeria jalzici* sp.nov.	Markov Ponor, Lipovo Polje, Lika, HR	M2		JX524675	JX524648	JX524702
		M6		JX524676	JX524649	JX524703
		M9	JX099468	JX099489	JX099447	JX099427
*Congeria jalzici* sp.nov.	Dankov Ponor, Lipovo Polje, Lika, HR	DP2		JX524662	JX524635	JX524689
		DP3	JX473583	JX524663	JX524636	JX524690
*Congeria jalzici* sp.nov.	Lukina Jama – Trojama Cave System, Northern Velebit, Lika, HR	L1	JX099466	JX099487	JX099445	JX099425
		L2		JX524673	JX524646	JX524700
		L3		JX524674	JX524647	JX524701
		L4	JX099467	JX099488	JX099446	JX099426
*Congeria jalzici* sp.nov.	Izvir Jamske Školjke, Metlika, Bela Krajina, SI	I1	JX099462	JX099483	JX099441	JX099421
		I2	JX099463	JX099484	JX099442	JX099422
		I3		JX524668	JX524641	JX524695
*Mytilopsis sallei*	Lam Tsuen River, Shatin, Hong Kong, China		JX099476	JX099497	JX099455	JX099435
			JX099477		JX099456	
*Mytilopsis leucophaeata*	GenBank		AF305704	EF414468	EF414448	HM100258
*Dreissena polymorpha*	Jarun Lake, Zagreb, HR		JX099478	JX099499	JX099458	JX099437
*Dreissena bugensis*	Ijsselmeer, Lelystadt, The Netherlands		JX099479	JX099498	JX099457	JX099436
*Dreissena presbensis*	GenBank		-	EF414469	EF414449	EF414491
*Dreissena blanci*	GenBank		-	EF414471	EF414459	EF414483
*Sphaerium* spp.	GenBank		*S. corneum*	*S. corneum*	*S. striatinum*	*S. striatinum*
			AM774537	AM779711	AF152041	AF120667
*Mya arenaria*	GenBank		AF120560	FM999792	AY377618	AF68
*Corbicula fluminea*	GenBank		AF120557	DQ343848	AF038999	U47647

### DNA sequence alignment

Sequences were aligned using the ClustalW option in BioEdit [39], and the resulting alignments were inspected manually and tested using Gblocks Server [40,41]. The regions identified as problematic and aligned poorly were excluded from subsequent analyses. The *COI* fragment showed significant variation, which was confirmed with the test for substitution saturation [42] implemented in Dambe [43]. Since there were no topological differences between the analyses ran with or without the 3^rd^ codon position, the COI 3^rd^ codon position was included in all phylogenetic analyses.

### Phylogenetic analyses

The final dataset consisted of 1736 bp of the *18S rRNA* gene, 1046 bp of the *28S rRNA*, 470 bp of the *16S rRNA* and 595 bp of the *COI* gene fragment. The best-fit partitioning schemes as well as nucleotide substitution models for each partition were calculated using the PartitionFinder [44]. “Branchlengths” were set to unlinked allowing branch length to be estimated independently for each subset, “search” to all in order to analyse all possible partitioning schemes, and “models” were set to mrbayes or to all, depending on whether the results were used for setting up Bayesian or ML analyses, respectively.

The rate heterogeneity test performed in PAUP [45] showed significant incongruence between different partitions. The phylogenetic analyses were, therefore, performed for each gene fragment separately. Inspection of the results revealed inconsistencies in the positions of *Dreissena* spp. in the *18S rRNA* tree and the position of *Mytilopsis* spp. in the *16S* gene tree. Since none of the conflicting branches had good posterior probabilities or bootstrap support, concatenated trees were constructed using Bayesian, MP and ML methods.

Maximum parsimony (MP) analyses were conducted in MEGA 5 [46]. The trees were obtained using the default settings. All sites were equally weighted and gaps were partially deleted (sites were deleted if gaps were present in more than 5% of the sequences). The resulting phylogeny was tested by 5,000 bootstrap replications.

Several independent Bayesian searches were run in MrBayes version 3.1.2 [47] for altogether 10 million generations with a sampling density of 1/100. The starting tree was random and partitioning scheme and substitution model type fixed according to results in PartitionFinder while model parameter values were estimated. First 17% of the generations that had average standard deviation of the split frequencies above 0,01 were discarded as burn in. Additionally, mixing of chains and ESS values were checked using Tracer [48]. Bayesian posterior probabilities (BPP) were estimated from the 50% majority-rule consensus tree.

Five replicates of maximum likelihood (ML) searches were performed in Garli [49]. Partitions and model types were set as determined in PartitionFinder while model parameter values were estimated by the program. All program settings were default except “streefname” which was set to random enabling multiple searches with random starting trees, “modweight” which was set to 0.003 according to developer’s instructions in order to ensure that partitioned models are properly optimised and during 200 bootstrap replicates “genthreshfortopoterm”, the first part of termination condition, was set to 10,000. Bootstrap consensus trees were obtained in Geneious Basic.

Divergence times were calculated on a reduced dataset using the relaxed clock in Beast 1.6.2. The dataset consisted of one sequence from the putative type locality of each *Congeria* lineage. *Mya arenaria* was included as an outgroup. The dataset was partitioned by different gene fragments, and substitution models were unlinked. To ensure that the dataset was robust enough for the divergence dating and that posterior ranges were not dominated by prior choices, we used both lognormal and exponential clocks, explored different prior distributions on various parameters (calibration nodes and means of the branch rates) and ran the analysis using sampling from the prior only. The Birth-death model was used as a tree prior.

Based on fossil data, we set up two points to calibrate the tree. The first undisputed dreissenid fossil is from the Priabonian Age (33.9–37.2 MYA), but there is also a questionable record from the Ypressian Age (48.6–55.8 MYA) [31]. We, therefore, used uniform prior spanning entire Eocene (55.8–33.9 MYA) to calibrate the family node. The origin of *Dreissena* was used as a second calibration point. *Dreissena* has a clear morphological feature, the lack of a shell apophysis for the attachment of the anterior byssal retractor muscle, which distinguishes it from other, both extant and fossil, genera. Although this feature has previously been considered a polyphyletic trait (references in Müller et al., 1999, e.g., Papp, 1950; 1985; Lueger, 1980; Taylor in Gray, 1988), our and other genetic studies [27,35] clearly demonstrate that *Dreissena* and, therefore, the loss of the apophysis, is of monophyletic origin. *Dreissena* first appeared in Lake Pannon 11.6 MYA [3]. Accordingly, we used lognormal prior with the onset of 11.6 MYA and a standard deviation of 0.75 to include the beginning of the Sarmatian Period (12.7 MYA) within the 95% of the prior probability density, because part of the Lake Pannon fauna, including dreissenids, originated in the Samartian Paratethyan Lakes [2,50].

With the final settings, we ran three independent runs and a total of 60 million generations, which after 15% burn in, yielded 51 million trees. We compared independent chains in Tracer to ensure that the chains had reached stationarity and converged to the same posterior distribution. There was no significant difference between the runs, and LogCombiner was thus used to pool all estimates into one file.

## Competing interests

The authors declare no competing interests.

## Authors’ contributions

HB conceived and designed the study, performed field work, carried out the molecular genetic studies, participated in the phylogenetic and morphological analysis, performed molecular clock analysis and drafted the manuscript. BM performed the morphological studies and statistical analysis and drafted the manuscript. MP participated in the phylogenetic analysis, HĆ conceived, designed and coordinated the study. All authors read and approved the manuscript.

## Supplementary Material

Additional file 1 **Shell morphometrics.** The file contains details of the methods and results of morphometric shell measurements. Click here for file

Additional file 2 **The PCR primers, reactions and conditions used in this study.** The file contains details on the PCR primers, reaction components and cycling conditions used in the study. Click here for file

## References

[B1] Morton B Velkovrh F Sket B Biology and anatomy of the “living fossil” *Congeria kusceri* (Bivalvia: Dreissenidae) from subterranean rivers and caves in the Dinaric karst of the former Yugoslavia J Zool 1998 245 147 174

[B2] Müller P Geary DH Magyar I The endemic molluscs of the Late Miocene Lake Pannon: their origin, evolution, and family-level taxonomy Lethaia 1999 32 47 60

[B3] Harzhauser M Mandic O van der Velde G, Rajagopal S, Bij de Vaate A Neogene dreissenids in Central Europe: evolutionary shifts and diversity changes The Zebra Mussel in Europe 2010 Weikersheim: Backhuys Publishers, Leiden/Margraf Publishers 11 29

[B4] Nuttall CP Review of the Caenozoic heterodont bivalve superfamily Dreissenacea Palaeontology 1990 33 707 737

[B5] Kochansky-Devide V Sliskovic T Miocenske kongerije Hrvatske, Bosne i Hercegovine 1978 Zagreb: Jugoslavenska Akademija Znanosti i Umjetnosti 19

[B6] Culver DC Sket B Hotspots of subterranean biodiversity in caves and wells J Cave Karst Stud 2000 62 11 17

[B7] Deharveng L Gibert J Culver DC White WB, Culver DC Diversity patterns in Europe Encyclopedia of Caves 2011 2 New York: Academic Press 219 228

[B8] Sket B High biodiversity in hypogean waters and its endangerment - The situation in Slovenia, the Dinaric Karst, and Europe Crustaceana 1999 72 767 779 10.1163/156854099503951

[B9] Sket B Presenetljive novosti v jamski favni Bosanske Krajine Naše jame 1970 11 93 99

[B10] Jalžić B The first finding of a live stygobiont bivalve *Congeria* in the Lika region, Croatia Nat Croat 2001 10 213 220

[B11] Cuttelod A Seddon M Neubert E European Red List of Non-Marine Molluscs 2011 Luxembourg: Publications Office of the European Union

[B12] Bilandžija H Jalžić B Ozimec R, Zagreb KL Dinaric cave clam, *Congeria kusceri* Bole Red Book of Croatian Cave Dwelling Fauna 2009 Republic of Croatia: Ministry of Culture, State Institute for Nature Protection 67 68

[B13] Culver DC Pipan T The biology of caves and other subterranean habitats 2009 USA: Oxford University Press

[B14] Bilandžija H Ćetković H Jeffery WR Evolution of albinism in cave planthoppers by a convergent defect in the first step of melanin biosynthesis Evol Dev 2012 14 196 203 10.1111/j.1525-142X.2012.00535.x 23017027 PMC6169799

[B15] Trontelj P Douady CJ Fišer C Gibert J Gorički Š Lefébure T Sket B Zakšek V A molecular test for cryptic diversity in ground water: how large are the ranges of macro-stygobionts? Freshwater Biol 2009 54 727 744 10.1111/j.1365-2427.2007.01877.x

[B16] Gorički Š Trontelj P Structure and evolution of the mitochondrial control region and flanking sequences in the European cave salamander *Proteus anguinus* Gene 2006 378 31 41 16764998 10.1016/j.gene.2006.04.016

[B17] Zakšek V Sket B Trontelj P Phylogeny of the cave shrimp *Troglocaris*: Evidence of a young connection between Balkans and Caucasus Mol Phylogenet Evol 2007 42 223 235 10.1016/j.ympev.2006.07.009 16935529

[B18] Zakšek V Sket B Gottstein S Franjević D Trontelj P The limits of cryptic diversity in groundwater: phylogeography of the cave shrimp *Troglocaris anophthalmus* (Crustacea: Decapoda: Atyidae) Mol Ecol 2009 18 931 946 10.1111/j.1365-294X.2008.04061.x 19207253

[B19] Verovnik R Sket B Trontelj P Phylogeography of subterranean and surface populations of water lice *Asellus aquaticus* (Crustacea: Isopoda) Mol Ecol 2004 13 1519 1532 10.1111/j.1365-294X.2004.02171.x 15140095

[B20] Stepien C Morton B Dabrowska K Guarnera R Radja T Radja B Genetic diversity and evolutionary relationships of the troglodytic “living fossil” *Congeria kusceri* (Bivalvia: Dreissenidae) Mol Ecol 2001 10 1873 1879 10.1046/j.0962-1083.2001.01329.x 11555232

[B21] Schütt H The taxonomical situation in the genus *Congeria* Partsch Proceedings of the Tenth International Malacological Congress 1991 Tubingen 607 610

[B22] Sket B Origins of the Dinaric troglobiotic mussel and its correct taxonomical classification. *Congeria* or *Mytilopsis* (Bivalvia: Dreissenidae)? Acta Biologica Slovenica 2012 54 67 76

[B23] Morton B Studies on the biology of *Dreissena polymorpha* Pall III. Population dynamics Proc Malacol Soc Lond 1969 38 471 482

[B24] Morton B Life-history characteristics and sexual strategy of *Mytilopsis sallei* (Bivalvia, Dreissenacea), introduced into Hong-Kong J Zool 1989 219 469 485 10.1111/j.1469-7998.1989.tb02594.x

[B25] Morton B Puljas S Life history strategy, with ctenidial and mantle larval brooding, of the troglodytic “living fossil” *Congeria kusceri* (Bivalvia: Dreissenidae) from the Dinaric karst of Croatia Biol J Linn Soc Lond 2013 108 294 314 10.1111/j.1095-8312.2012.02020.x

[B26] Stepien CA Hubers AN Skidmore JL Diagnostic genetic markers and evolutionary relationships among invasive dreissenoid and corbiculoid bivalves in North America: phylogenetic signal from mitochondrial 16S rDNA Mol Phylogenet Evol 1999 13 31 49 10.1006/mpev.1999.0666 10508537

[B27] Stepien CA Taylor CD Grigorovich IA Shirman SV Wei R Korniushin AV Dabrowska KA DNA and systematic analysis of invasive and native dreissenid mussels: Is *Dreissena bugensis* really *D rostriformis?* Aquat Invaders 2003 14 8 18

[B28] Drummond AJ Ho SYW Phillips MJ Rambaut A Relaxed phylogenetics and dating with confidence PLoS Biol 2006 4 e88 10.1371/journal.pbio.0040088 16683862 PMC1395354

[B29] Drummond A Rambaut A BEAST: Bayesian evolutionary analysis by sampling trees BMC Evol Biol 2007 7 214 10.1186/1471-2148-7-214 17996036 PMC2247476

[B30] Rögl F Mediterranean and Paratethys. Facts and hypotheses of an Oligocene to Miocene paleogeography (short overview) Geol Carpathica 1999 50 339 349

[B31] Benton MJ Fossil Record 2 1993 London: Chapman & Hall

[B32] Wilke T How dependable is a non-local molecular clock? A reply to Hausdorf et al. (2003) Mol Phylogenet Evol 2004 30 835 840 10.1016/j.ympev.2003.08.008 15012962

[B33] Wilke T Schultheiß R Albrecht C Bornmann N Trajanovski S Kevrekidis T Native *Dreissena* freshwater mussels in the Balkans: in and out of ancient lakes Biogeosciences 2010 7 3051 3065 10.5194/bg-7-3051-2010

[B34] Trontelj P Gorički S Polak S Verovnik R Zakšek V Sket B Age estimates for some subterranean taxa and lineages in the Dinaric Karst Acta Carsologica 2007 36 183 189

[B35] Albrecht C Schultheiß R Kevrekidis T Streit B Wilke T Invaders or endemics? Molecular phylogenetics, biogeography and systematics of *Dreissena* in the Balkans Freshwater Biol 2007 52 1525 1536 10.1111/j.1365-2427.2007.01784.x

[B36] Verovnik R Sket B Trontelj P The colonization of Europe by the freshwater crustacean *Asellus aquaticus* (Crustacea: Isopoda) proceeded from ancient refugia and was directed by habitat connectivity Mol Ecol 2005 14 4355 4369 10.1111/j.1365-294X.2005.02745.x 16313598

[B37] Park J-K O’Foighil D Sphaeriid and corbiculid clams represent separate heterodont bivalve radiations into freshwater environments Mol Phylogenet Evol 2000 14 75 88 10.1006/mpev.1999.0691 10631043

[B38] Taylor JD Williams ST Glover EA Dyal P A molecular phylogeny of heterodont bivalves (Mollusca: Bivalvia: Heterodonta): new analyses of 18S and 28S rRNA genes Zool Scr 2007 36 587 606 10.1111/j.1463-6409.2007.00299.x

[B39] Hall TA BioEdit: a user-friendly biological sequence alignment editor and analysis program for Windows 95/98/NT Nucleic acids symposium series 1999 95 98 Volume 4110780396

[B40] Castresana J Selection of conserved clocks from multiple alignments for their use in phylogenetic analysis Mol Biol Evol 2000 17 540 552 10.1093/oxfordjournals.molbev.a026334 10742046

[B41] Talavera G Castresana J Improvement of phylogenies after removing divergent and ambiguously aligned blocks from protein sequence alignments Syst Biol 2007 56 564 577 10.1080/10635150701472164 17654362

[B42] Xia X Xie Z Salemi M Chen L Wang Y An index of substitution saturation and its application Mol Phylogenet Evol 2003 26 1 7 10.1016/S1055-7903(02)00326-3 12470932

[B43] Xia X Xie Z DAMBE: software package for data analysis in molecular biology and evolution J Hered 2001 92 371 373 10.1093/jhered/92.4.371 11535656

[B44] Lanfear R Calcott B Ho SYW Guindon S PartitionFinder: combined selection of partitioning schemes and substitution models for phylogenetic analyses Mol Biol Evol 2012 29 1695 1701 10.1093/molbev/mss020 22319168

[B45] Swofford D PAUP*. Phylogenetic Analysis Using Parsimony (*and Other Methods). Version 4 2003 Sunderland, MA: Sinauer Associates

[B46] Tamura K Peterson D Peterson N Stecher G Nei M Kumar S MEGA5: molecular evolutionary genetics analysis using maximum likelihood, evolutionary distance, and maximum parsimony methods Mol Biol Evol 2011 28 2731 2739 10.1093/molbev/msr121 21546353 PMC3203626

[B47] Ronquist F Huelsenbeck JP MrBayes 3: Bayesian phylogenetic inference under mixed models Bioinformatics 2003 19 1572 1574 10.1093/bioinformatics/btg180 12912839

[B48] Rambaut A Drummond AJ Tracer v1. 4 2007 Available from http://beast.bio.ed.ac.uk/Tracer

[B49] Zwickl DJ Genetic algorithm approaches for the phylogenetic analysis of large biological sequence datasets under the maximum likelihood criterion. PhD thesis 2006: The University of Texas at Austin

[B50] Harzhauser M Mandic O Neogene lake systems of Central and South-Eastern Europe: Faunal diversity, gradients and interrelations Palaeogeogr Palaeoclimatol Palaeoecol 2008 260 417 434 10.1016/j.palaeo.2007.12.013

