# Supplementary material for: Evolutionary history of relict Congeria (Bivalvia: Dreissenidae): unearthing the subterranean biodiversity of the Dinaric Karst
Source: Front Zool. 2013 Feb 6;10:5. doi: 10.1186/1742-9994-10-5 (PMC3599595; doi:10.1186/1742-9994-10-5)
Supplement: Additional file 1 — Shell morphometrics. The file contains details of the methods and results of morphometric shell measurements. [file 1742-9994-10-5-S1.pdf]

Additional file 1

Shell morphometrics

Results

Morphometric shell measures differed between the three putative species of *Congeria* (Table S1). Differences in the median values of shell length among the three species groups were significant (H = 0.238; df = 2; $P = 0.010$). Both pairwise median shell length and median shell width of *C. mulaomerovici* sp. nov. differed significantly from that of the two sister species, whereas *C. jalzici* sp. nov. and *C. kusceri* median shell lengths and widths were not significantly different from each other ($p > 0.05$). Overall shell height medians were significantly different. Median shell height of *C. mulaomerovici* sp. nov. differed significantly from *C. kusceri* but not *C. jalzici* sp. nov., and nor did *C. jalzici* sp. nov. differ from *C. kusceri*. Shell width median differences were highly significant.

The results of linear regression undertaken separately for each of the putative species of *Congeria* indicated that length to height, and height to width length have a very good linear predictability to each other ($r^2 > 0.8$).

Comparison of species specific shell morphometrics by general linear modelling of the linear regression lines of shell length (SL) as a function of shell height (SH), shell length (SL) of shell width (SW) and shell height (SH) of shell width (SW) were not significantly different with regard to their slopes. Comparison of linear regression line intercepts, however, showed that *C. jalzici* sp. nov. deviated significantly from *C. kusceri* and *C. mulaomerovici* sp. nov., whereas the intercept difference between *C. kusceri* and *C. mulaomerovici* sp. nov. was not significant ($p > 0.05$) (Fig.S1).

Methods

Intact shells of all *Congeria* specimens were measured using Vernier calipers along their greatest length and maximum heights and widths to the nearest 0.5 mm. These datasets were pooled for an analysis of shell dimension differences between populations and putative species and examined for statistically significant differences. Because the normality test for differences in One Way Annova did not support significant differences for all three shell dimensions, an overall comparison between groups was made using a Kruskal-Wallis One Way ANOVA on Ranks, and all Pairwise Multiple Comparison Procedures using Dunn's Method. Comparisons of species-specific shell dimensions were made using a general linear modeling of regression lines (linear), slope and intercept. The general linear modelling was carried out using RStudio (v. 0.95.262) to test for differences in slope and intercept of regression lines of the three putative species [1]. Tests were carried out using Sigmastat.

1. Zuur AF, Ieno EN, Smith GM: *Analysing ecological data*. Springer New York; 2007.

Figures

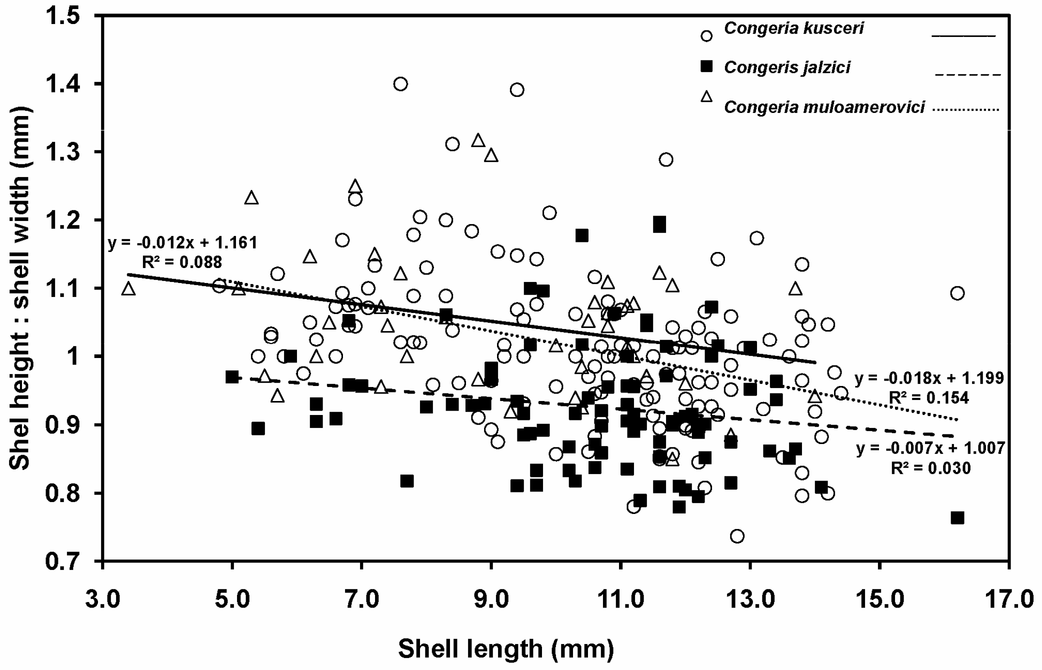

Figure S1 - Shell morphometrics of the three putative species of Congeria.

Congeria kusceri (O), *Congeria jalzici* sp. nov. (●) and *Congeria mulaomerovici* sp. nov. (Δ).

Linear correlations between shell length and shell height/shell width are low ($r^2 < 0.2$).

Tables

Table S1 - Statistical tests of differences in morphometric shell measures between the three putative species of Congeria.

An overall comparison of medians was tested using Kruskal-Wallis One Way ANOVA on Ranks, and all Pairwise Multiple Comparison Procedures using Dunn's Method. Significance levels are indicated by: *** = $p \leq 0.001$; ** = $p \leq 0.01$; * = $p \leq 0.05$; and ns = not significant.

	**SL, mm**		**SH, mm**		**SW, mm**	
**Overall comparisons of median**	H	*P*	H	*P*	H	*P*
	0.238	**	8.245	*	14.306	***
**Pairwise comparisons of median**	Q	*p*	Q	*p*	Q	*p*
C. kusceri vs. *C. jalzici*	-	ns	-	ns	-	ns
C. kusceri vs. *C. mulaomerovici*	2.772	*	2.790	*	3.182	*
C. jalzici vs. *C. mulaomerovici*	2.862	*	-	ns	3.710	*
