# Supplementary material for: Evolutionary history of relict Congeria (Bivalvia: Dreissenidae): unearthing the subterranean biodiversity of the Dinaric Karst
Source: Front Zool. 2013 Feb 6;10:5. doi: 10.1186/1742-9994-10-5 (PMC3599595; doi:10.1186/1742-9994-10-5)
Supplement: Additional file 2 — The PCR primers, reactions and conditions used in this study. The file contains details on the PCR primers, reaction components and cycling conditions used in the study. [file 1742-9994-10-5-S2.pdf]

Additional file 2

The PCR primers, reactions and conditions used in this study.

Region	Primers	Primer sequence	Primer reference	PCR conditions	PCR reaction
COI	HCO2198	taaacttcagggtgaccaaaaaatca	[57]	94ºC 5min<br>94ºC 30s<br>50ºC 30s 35x<br>72ºC 40s<br>72ºC 5min	25 µL PCR mix*<br>15 pmol each primer<br>80-100 ng DNA<br>35-40 pmol Mg<br>water to 50 µL
	COL1	ttgtgrgctggcttggtg	This study		
*18S***	18SKBPF	ctggtgccagcagccgcgg	[58]	94ºC 5min<br>94ºC 30s<br>50ºC 45s 35x<br>72ºC 70s<br>72ºC 5min	25 µL PCR mix<br>20 pmol each primer<br>80-100 ng DNA<br>water to 50 µL
	18SKBPR	tggtgcccttccgtcaattcc			
	18SMR	ttgatccttctgcaggttcac	[59]		
	18SMF	aacctggttgatcctgccag			
28S+	D1F	gggactaccccctgaatttaagcat	[41]	94ºC 3min<br>94ºC 30s<br>TA++ 30s<br>72ºC 60s<br>72ºC 5min	25 µL PCR mix<br>15 pmol each primer<br>80-100 ng DNA<br>35-40 pmol Mg<br>50 ng BSA<br>water to 50 µL
	D2F	tcagtaagcggaggaa			
	D23F	gagagttcaagagtacgtg			
	D4RB	tgttagactccttggtccgtgt			
	D6R	ccagctatcctgagggaaacttcg			

	D6Rb	ggttccctccgaagtttcc	This study		
16S	16SLRN<br>16SLRJ	cgcctgtttatcaaaaacat<br>ctccggtttgaactcagatca	[60]	94ºC 2min<br>94ºC 30s<br>50ºC 30s 30x<br>72ºC 60s<br>72ºC 5min	25 µL PCR mix<br>15 pmol each primer<br>80-100 ng DNA<br>35-40 pmol Mg<br>water to 50 µL

* ReadyMix Taq PCR Reaction Mix with $MgCl_2$ (Sigma)

** The *18S rRNA* marker was amplified in two overlapping fragments, one with 18sKBPF/18sMR and the other with 18sMF/18sKBPR primer pairs.

[+] The *28S* gene fragment was amplified with either the most external primer pair (D1F/D6R) or with different combinations of primers.

[++] The Touch down PCR program was used to amplify the *28S rRNA* fragment. The annealing temperature was set at 52ºC during the first five cycles, 55ºC during the next five cycles and 58ºC for the last 25 cycles.
